# Effect of Notch signal pathway on H9c2 cardiomyocytes apoptosis induced by hypoxia/reoxygenation via ROCK2

**DOI:** 10.1186/1479-5876-10-S2-A62

**Published:** 2012-10-17

**Authors:** Yang Shen, Yun Guo Zhou, Xiao Gang Peng, Qing Cao, Kui Hong

**Affiliations:** 1 Cardiovascular section, the second affiliated of hospital to Nanchang University, Nanchang, 330006, China; 2 The Key Laboratory of Molecular Medicine, The Second Affiliated Hospital of Nanchang University, Nanchang, 330006, China

## Background

It is very important to explore the new strategies of prevention and treatment of Ischemic heart disease (IHD) in molecular level. Notch signaling pathways are very expedient in terms of the protection and recovery of myocardial when the heart muscle is impaired. It is also known that ROCKs are closely related to the apoptosis of cardiomyocytes. We infer that there are interactions between Notch signaling pathways and ROCKs on cell apoptosis in H9c2 cardiomyocytes model of H/R.

## Objective

1. To investigate the effects of Notch signaling pathway on hypoxia/ reoxygenation (H/R) induced apoptosis of H9c2 cardiomyocytes in rat. 2. To explore the interaction between ROCK2 and NICD on H/R and then to reveal the mechanisms of Notch signaling pathway involved in the modulation of apoptosis and recovery of H9c2 cardiomyocytes model of H/R .

## Methods

1. The total length of cDNA fragment encoded with NICD was obtained from H9c2 cardiomyocytes from rat by reversing transcription polymerase chain reaction (RT-PCR), then the cDNA fragment was inserted into pCMV-Tag2B vector. The recombinant plasmid was confirmed by restriction endonuclease (EcoRI and SalI) digestion and DNA sequencing. H9c2 cardiomyocytes were transfected by pCMV-Tag2B-NICD and the expression of NICD was detected by Western blot. 2. The cultured H9c2 cardiomyocytes were randomly divided into six groups: Control group; H/R group; NICD group; NICD+H/R group; DAPT group; DAPT+H/R group. Apoptosis of each group was analyzed by flow cytometry (FCM), and Western blot was applied to assess the expression of ROCK2 and NICD proteins.

## Results

1. The recombinant plasmid pCMV-Tag2B-NICD was successfully constructed. After transfection into H9c2 cardiomyocytes, Western blot analysis showed that NICD was highly expressed in H9c2 cardiomyocytes. 2. The overexpression of NICD results in a rise of protein expression of NICD, and reduces the protein expression of ROCK2 and apoptosis of H9c2 cardiomyocytes; on the contrary, the supression of NICD leads to a reduction of protein expression of NICD, and increases the protein expression of ROCK2 and apoptosis of H9c2 cardiomyocytes.

## Conclusion

1. The recombinant plasmid pCMV-Tag2B-NICD was successfully constructed. 2. After hypoxia/reoxygenation, the protein expression of NICD was significantly increased, and the protein expression of ROCK2 was decreased, compared to control group. 3. Notch signaling pathway reduced the H/R-induced apoptosis of rat H9c2 cardiomyocytes via supression of ROCK2 from the rat.

